# Improving preparedness to respond to cross-border hepatitis A outbreaks in the European Union/European Economic Area: towards comparable sequencing of hepatitis A virus

**DOI:** 10.2807/1560-7917.ES.2019.24.28.1800397

**Published:** 2019-07-11

**Authors:** Theresa Enkirch, Ettore Severi, Harry Vennema, Lelia Thornton, Jonathan Dean, Maria-Louise Borg, Anna Rita Ciccaglione, Roberto Bruni, Iva Christova, Siew Lin Ngui, Koye Balogun, Vratislav Němeček, Mia Kontio, Mária Takács, Andrea Hettmann, Rita Korotinska, Arthur Löve, Ana Avellón, Milagros Muñoz-Chimeno, Rita de Sousa, Denisa Janta, Jevgenia Epštein, Sofieke Klamer, Vanessa Suin, Stephan W Aberle, Heidemarie Holzmann, Kassiani Mellou, Josefine Lundberg Ederth, Lena Sundqvist, Anne-Marie Roque-Afonso, Sanja Kurečić Filipović, Mario Poljak, Line Vold, Kathrine Stene-Johansen, Sofie Midgley, Thea Kølsen Fischer, Mirko Faber, Jürgen J Wenzel, Johanna Takkinen, Katrin Leitmeyer

**Affiliations:** 1 Public Health Agency of Sweden, Solna, Sweden; 2 European Programme for Public Health Microbiology Training (EUPHEM), European Centre for Disease Prevention and Control (ECDC), Solna, Sweden; 3 European Centre for Disease Prevention and Control (ECDC), Solna, Sweden; 4 Karolinska Institutet, Stockholm, Sweden; 5 National Institute for Public Health and the Environment (RIVM), Bilthoven, the Netherlands; 6 HSE - Health Protection Surveillance Centre, Dublin, Ireland; 7 National Virus Reference Laboratory, Dublin, Ireland; 8 Ministry of Health, Msida, Malta; 9 National Institute of Health, Rome, Italy; 10 National Center of Infectious and Parasitic Diseases, Sofia, Bulgaria; 11 Public Health England (PHE), London, United Kingdom; 12 National Institute of Public Health, Prague, Czech Republic; 13 National Institute for Health and Welfare (THL), Helsinki, Finland; 14 National Public Health Institute, Budapest, Hungary; 15 Centre for Disease Prevention and Control, Riga, Latvia; 16 Landspitali- National University Hospital, Reykjavik, Iceland; 17 Carlos III Institute of Health, Madrid, Spain; 18 National Institute of Health Dr. Ricardo Jorge (INSA), Lisbon, Portugal; 19 National Institute of Public Health, Bucharest, Romania; 20 Health Board, Tallinn, Estonia; 21 Scientific Institute of Public Health, Brussels, Belgium; 22 Sciensano, Directorate Infectious diseases in humans, Brussels, Belgium; 23 Center for Virology, Medical University of Vienna, Vienna, Austria; 24 Hellenic Centre for Disease Control and Prevention, Athens, Greece; 25 National Reference Centre for Hepatitis A, Villejuif, France; 26 Croatian Institute of Public Health, Zagreb, Croatia; 27 Institute of Microbiology and Immunology, Ljubljana, Slovenia; 28 Norwegian institute of Public Health, Oslo, Norway; 29 Statens Serum Institut (SSI), Copenhagen, Denmark; 30 Department of Infectious Diseases and Global Health, University of Southern Denmark, Odense, Denmark; 31 Robert Koch Institute (RKI), Berlin, Germany; 32 National Reference Laboratory for HAV, Regensburg University Medical Center, Regensburg, Germany

**Keywords:** hepatitis A virus, HAV, sequence analysis, European Union, surveys and questionnaires, foodborne diseases, capacity building

## Abstract

**Introduction:**

Sequence-based typing of hepatitis A virus (HAV) is important for outbreak detection, investigation and surveillance. In 2013, sequencing was central to resolving a large European Union (EU)-wide outbreak related to frozen berries. However, as the sequenced HAV genome regions were only partly comparable between countries, results were not always conclusive.

**Aim:**

The objective was to gather information on HAV surveillance and sequencing in EU/European Economic Area (EEA) countries to find ways to harmonise their procedures, for improvement of cross-border outbreak responses.

**Methods:**

In 2014, the European Centre for Disease Prevention and Control (ECDC) conducted a survey on HAV surveillance practices in EU/EEA countries. The survey enquired whether a referral system for confirming primary diagnostics of hepatitis A existed as well as a central collection/storage of hepatitis A cases’ samples for typing. Questions on HAV sequencing procedures were also asked. Based on the results, an expert consultation proposed harmonised procedures for cross-border outbreak response, in particular regarding sequencing. In 2016, a follow-up survey assessed uptake of suggested methods.

**Results:**

Of 31 EU/EEA countries, 23 (2014) and 27 (2016) participated. Numbers of countries with central collection and storage of HAV positive samples and of those performing sequencing increased from 12 to 15 and 12 to 14 respectively in 2016, with all countries typing an overlapping fragment of 218 nt. However, variation existed in the sequenced genomic regions and their lengths.

**Conclusions:**

While HAV sequences in EU/EEA countries are comparable for surveillance, collaboration in sharing and comparing these can be further strengthened.

## Introduction

The hepatitis A virus (HAV) of the *Picornaviridae* family affects 114 million people annually and is a common cause of food-borne infections worldwide [1,2]. It is mainly transmitted through faecal-oral route via person-to-person contact or contaminated food and water [3]. Symptoms range from fever, nausea, diarrhoea, abdominal pain and jaundice to acute liver failure. In children however, the majority of HAV infections are subclinical and asymptomatic [4].

Hepatitis A is a notifiable disease in all countries of the European Union/European Economic Area (EU/EEA) [5]. Most EU/EEA countries, with the exception of few eastern EU countries, have experienced hepatitis A notification rates below two cases per 100,000 population in the last two decades or more [6]. Consequently, and based on World Health Organization (WHO) classification, the HAV endemicity is currently low or very low with a resulting high population susceptibility [1,7]. In line with WHO guidance, in most EU/EEA countries with low or very low endemicity, hepatitis A vaccination is recommended only to individuals at increased risk of infection, including travellers to endemic countries, people who inject drugs (PWID) and men who have sex with men (MSM). However, due to suboptimal vaccination uptakes, travel to countries with high or intermediate HAV endemicity is a common risk factor for infection [8] and multi-country outbreaks have been reported since 2012 among MSM and PWID [9,10]. Starting 2016, a large outbreak disproportionally affecting MSM was identified in Europe [11-13]. Between June 2016 and May 2017, ca 1,400 hepatitis A cases in 16 EU/EEA countries were identified, with genome sequencing showing concomitant circulation of three genetically distinct HAV strains [14,15]. In the same period, several other HAV outbreaks affecting MSM were reported from the United States, Israel and Chile [16]. In recently published studies, sequencing demonstrated a link between the cases in Israel and Chile and the ongoing EU outbreak [17,18]. Also a large food-borne outbreak caused by berries was reported in the EU/EEA in 2013–14, with sequencing being key in identifying the vehicle of infection [19,20].

Sequence-based typing together with conventional epidemiological methods play a crucial role in outbreak investigations by confirming or refuting transmission chains, monitoring circulating strains and documenting introduction of new strains [20]. The combined molecular and epidemiological methods provide important information for action, allowing rapid implementation of public health measures such as vaccination of risk groups or identification of contaminated vehicles of infection, which can be traced back and forward and, when these are commercialised, taken off the market to avoid new infections. To respond to multi-country hepatitis A outbreaks, similar sequencing methods (same amplified target region, including length, for sequencing and phylogenetic analysis) and collaborations and exchange of HAV sequence information between EU/EEA countries offer substantial benefits. This was demonstrated during the large 2013–14 EU-wide outbreak related to frozen berries, although sequencing results were not always comparable as different amplified regions of the HAV genome were used for typing in some of the countries [19,20].

The HAV genome consists of linear positive single-stranded RNA and has a single open reading frame (ORF) divided into three functional regions, P1–P3. P1 encodes the capsid polypeptides (viral protein VP1–4) and P2 and P3 encode the non-structural polyproteins 2A–2C and 3A–3D required for virus replication. Only one single serotype has been described but six HAV genotypes are defined. The genotypes differ by 15–25% in the nt sequence of the VP1/2A region and are further divided into subtypes (7.0–7.5% difference). Subtypes A and B of genotype I, II and III are infectious for humans [21-24]. In Europe, subgenotype IA is the most predominant subgenotype [25]. The presence of only one known serotype can partially be explained by the low mutation rate of HAV and the low variability resulting in a well-conserved polyprotein [26].

After the large 2013–14 berry-related EU-wide outbreak, the recognition of the importance of sequencing in terms of outbreak investigations and preparedness led ECDC to convene in October 2014 a multidisciplinary expert meeting. The aim was to discuss the need for agreement on a common genomic region for sequencing and/or the possible use of a protocol that HAVNET, a global network of hepatitis A laboratories for sharing molecular and epidemiological data on hepatitis A cases [27], had developed. In preparation of this meeting, ECDC performed a survey among the EU/EEA countries in order to collect information on HAV genotyping practices, including whether primary diagnostic samples of patients with hepatitis A could be referred for confirmation or genotyping and whether such samples were centrally collected. The survey also aimed to identify the most suitable common target region for sequencing, and the existence of collaborations between the public health sector with HAVNET and with the food sector. Based on the findings of this first survey, it was agreed to recommend the sequencing of a common consensus sequencing region, spanning the HAV VP1/2A junction, and thus promoting the sequence target region described in the HAVNET protocol [27]. In 2016, ECDC followed-up with a second survey to assess change in sequencing and some of the surveillance practices over time. Our study describes the analysis of the results from these surveys and assesses the progress made between 2014 and 2016 in harmonisation of HAV typing practices.

## Methods

### Questionnaires

In September 2014, ECDC performed a rapid consultation among the 31 EU/EEA countries to mainly collect information on HAV genotyping practices through the National Focal Points for food- and waterborne diseases and zoonoses (Supplement 1). The results of this first online survey were discussed in the multidisciplinary HAV expert consultation meeting at ECDC in October 2014, where it was agreed to recommend a common consensus sequencing region and/or a standard protocol for HAV sequencing as proposed by HAVNET [27]. HAVNET provides a laboratory protocol for the detection and sequencing of HAV, which includes instructions on RNA extraction, PCR settings, primers for the amplification of the VP1/2A junction, and reagents that can be used. In August 2016, ECDC distributed a follow-up questionnaire (Supplement 2) to assess if practices had changed or had become more compatible between countries.

The responses to both questionnaires were requested by October 2014 and October 2016, respectively.

Both surveys focused on laboratory methods used at the national level for sequence-based typing of HAV. In 2014, the sequence target region for typing and/or the protocol used for sequencing were queried. Although these were not initially investigated by the 2016 survey, several countries nonetheless provided information about their sequencing protocol or sequence target region. To complete knowledge on this, countries that had not covered this topic were contacted via email after the deadline of the 2016 survey.

The surveys also included questions about molecular surveillance practices related to sequence-based typing. These questions aimed at gaining knowledge on the central collection and storage of diagnostic samples of patients fulfilling the EU hepatitis A case definition (IgM or HAV RNA positive, with clinical criteria of an acute hepatitis) [28], and on the presence of a referral system for confirmation of primary diagnostics or for characterisation by sequencing (e.g. genotyping). Furthermore, countries were asked about their sequencing sampling strategy during outbreak situations.

Further questions in both surveys were on collaborations with the food sector on comparing sequences obtained from humans and/or food samples (food safety authorities and reference laboratories for food) and on collaborations with HAVNET in depositing and/or comparing data in the HAVNET database.

In the 2016 survey, additional information was collected on the timeliness of sequence submission to HAVNET in 2015.

### Ethical statement

Ethical approval was not necessary for this study.

### Data analysis

Counts and proportions of country responses to survey questions were calculated using Excel. Maps were generated using ECDC Map Maker (EMMa) [29]. Alignments of the amplified regions of the HAV genome were done using CLC Genomics and the complete HAV genome sequence (GenBank accession number: NC_001489) was used as reference. A schematic representation of the alignment was drawn using PowerPoint.

We present a descriptive analysis of the responses to the surveys and compare the sequence-based typing practices for HAV reported by EU/EEA countries in 2014 and 2016.

## Results

### Survey participation

**Figure 1:**
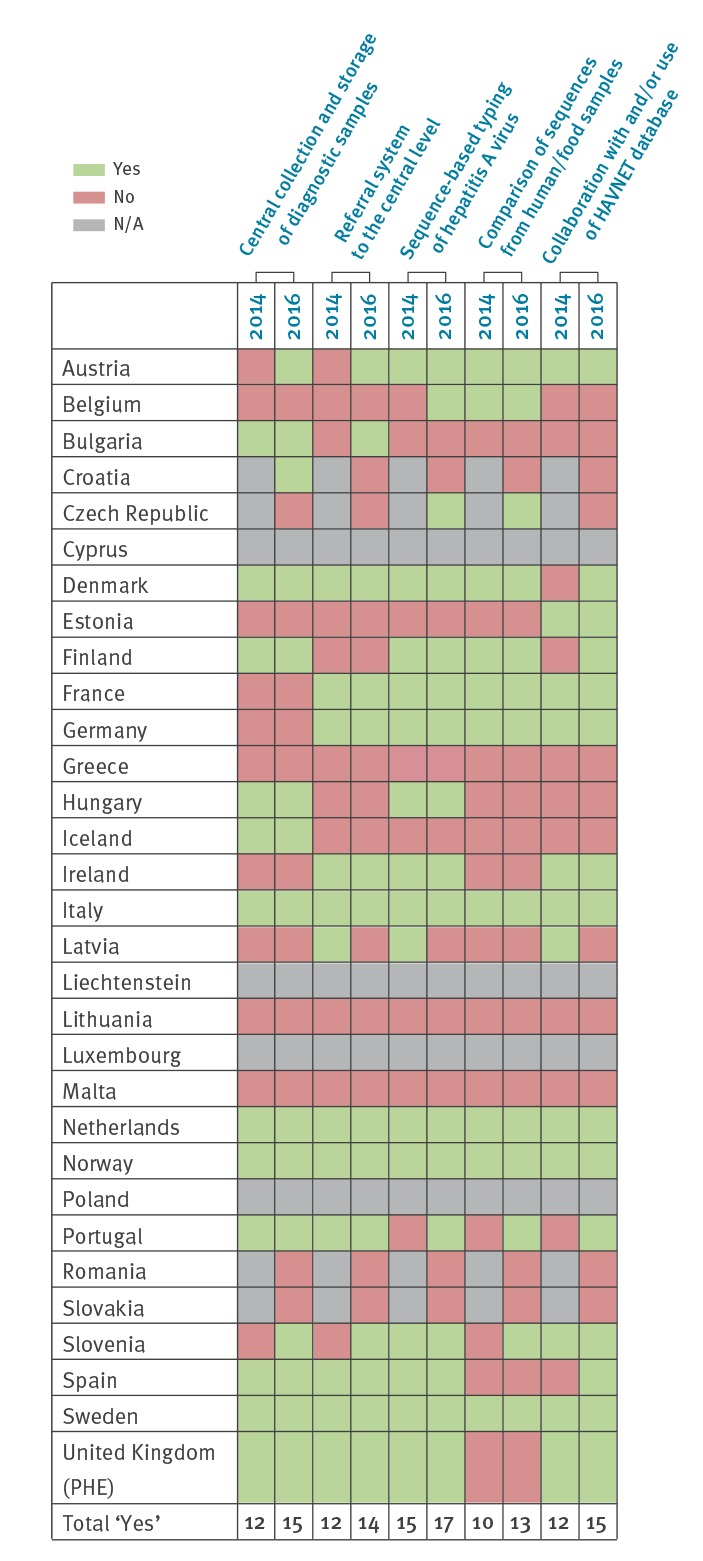
Overview of responses to surveyed questions by country, 2014 and 2016

PHE: Public Health England; UK: United Kingdom.

In 2014, 23 of 31 EU/EEA countries (74%) responded to the survey. In 2016, four more countries (Croatia, Czech Republic, Romania, Slovakia) replied to the survey (27/31) resulting in a participation of 87%. In both years, replies for the United Kingdom (UK) were provided by Public Health England and related to the practices ongoing in England, Northern Ireland and Wales. An overview of the countries participating in the surveys and procedural changes implemented over time is represented in Figure 1, with a total of 13 such changes visible.

### Sample collection, storage and referral system

In 2014, 12 of 23 countries indicated central collection and storage of their samples defined as HAV positive by primary diagnostics and intended for sequencing, mostly at national level. Twelve countries had a referral system for the confirmation of the primary diagnostics or for characterisation by sequencing which was voluntary in 10 and mandatory in two countries (one in outbreak situation only).

The number of countries with central collection and storage of HAV positive samples was 15 in 2016, whereas the number of countries with a referral system for confirmation of samples increased by two (n = 14) compared to 2014. Eleven countries reported a voluntary and three a mandatory system (one in outbreak situation only).

Austria and Slovenia reported no central collection and storage of HAV positive samples in 2014 but did so by the time of the second survey in 2016, as did Croatia, which had not participated in the 2014 survey. Austria, Bulgaria and Slovenia did not have a referral system in 2014 but all three countries established one by 2016. In contrast, Latvia had a referral system in 2014 with voluntary reporting but it was not the case any longer in 2016. In most countries, the national reference laboratories have a supporting role offering laboratory diagnostics or genotyping on request (e.g. Germany, Ireland, the Netherlands and Spain). In France, referral to the reference laboratory is mandatory only when clusters of cases or outbreaks are identified (Table).

**Table:** Central sample collection and place of storage, presence of a referral system for hepatitis A virus by country, European Union/European Economic Area, 2014 (n = 23 countries providing information) and 2016 (n = 27 countries providing information)

Countries	Central sample collection and place of storage		Presence of a referral system	
	2014 (n = 12/23)	2016 (n = 15/27)	2014 (n = 12/23)	2016 (n = 14/27)
Austria	No	National reference laboratory at the Center for Virology, Medical University of Vienna, Vienna	No	Yes, mandatory
Belgium	No	No	No	No
Bulgaria	National reference laboratory at the National Centre of Infectious and Parasitic Diseases, Sofia	National reference laboratory at the National Centre of Infectious and Parasitic Diseases, Sofia	No	Yes, voluntary
Croatia	NA	University Hospital for Infectious Diseases, Zagreb^a^	NA	No
Czech Republic	NA	No	NA	No
Denmark	Virus Surveillance and Research section at Statens Serum Institute, Copenhagen	Virus Surveillance and Research section at Statens Serum Institute, Copenhagen	Yes, voluntary	Yes, voluntary
Estonia	No	No	No	No
Finland	National Institute of Health and Welfare, Helsinki	National Institute of Health and Welfare, Helsinki	No	No
France	No	No	Yes, voluntary but mandatory during outbreaks/detection of clusters (2014 and 2016)	
Germany	No	No	Yes, voluntary	Yes, voluntary
Greece	No	No	No	No
Hungary	National Public Health Institute, Budapest	National Public Health Institute, Budapest	No	No
Iceland	Department of Virology, Landspitali - National University Hospital, Reykjavik	Department of Virology, Landspitali - National University Hospital, Reykjavik	No	No
Ireland	No	No	Yes, voluntary	Yes, voluntary
Italy	National Reference Laboratory of the Istituto Superiore di Sanità, Rome	National Reference Laboratory of the Istituto Superiore di Sanità, Rome	Yes, voluntary	Yes, voluntary
Latvia	No	No	Yes, voluntary	No
Lithuania	No	No	No	No
Malta	No	No	No	No
Netherlands	National public health institute, Bilthoven	National public health institute, Bilthoven	Yes, voluntary	Yes, voluntary
Norway	Norwegian Institute of Public Health, Oslo	Norwegian Institute of Public Health, Oslo	Yes, mandatory	Yes, mandatory
Portugal	The National Institute of Health, Lisbon	The National Institute of Health, Lisbon	Yes, voluntary	Yes, voluntary
Romania	NA	No	NA	No
Slovakia	NA	No	NA	No
Slovenia	No	Institute of Microbiology and Immunology, Faculty of Medicine, University of Ljubljana, Ljubljana	No	Yes, voluntary
Spain	National Center of Microbiology, Institute of Health Carlos III, Madrid	National Center of Microbiology, Institute of Health Carlos III, Madrid	Yes, voluntary	Yes, voluntary
Sweden	Public Health Agency of Sweden (PHAS), Solna^a^ and/or Sahlgrenska hospital, Gothenburg^a^	Public Health Agency of Sweden (PHAS), Solna^a^ and/or Sahlgrenska hospital, Gothenburg^a^	Yes, voluntary	Yes, voluntary
United Kingdom (PHE)	Virus Reference Department, PHE, Colindale	Virus Reference Department, PHE, Colindale	Yes, voluntary	Yes, voluntary

NA: data not available; PHE: Public Health England.

^a^ Only those samples that were also tested there by primary diagnostic or sequencing.

### Sequencing practices in European Union/European Economic Area countries

**Figure 2:**
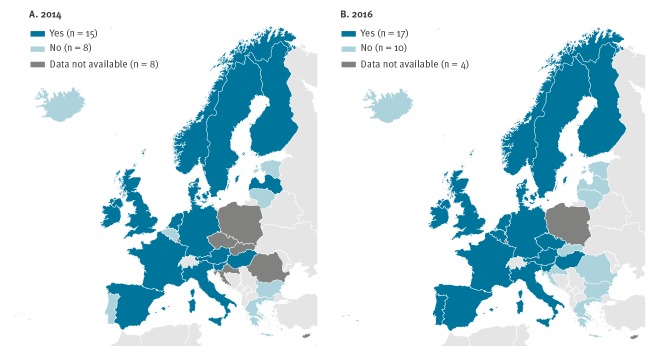
European Union/European Economic Area countries performing sequence-based typing of hepatitis A virus in (A) 2014 (n = 23 countries providing information) and (B) 2016 (n = 27 countries providing information)

More than half of the EU/EEA countries performed sequence-based typing (15/23 in 2014 and 17/27 in 2016) at their reference laboratory or collaborating centre (Figure 2A and B). Latvia performed sequencing in 2014 but stopped it by 2016. While no information was obtained for the Czech Republic in 2014, it was confirmed during the second survey in 2016 that sequencing had been implemented in this country after 2014. Thus a total of three countries (Belgium, the Czech Republic and Portugal) started sequence-based typing after 2014. Of eastern EU/EEA countries, the Czech Republic and Hungary reported having capacity for sequencing and its implementation.

**Figure 3:**
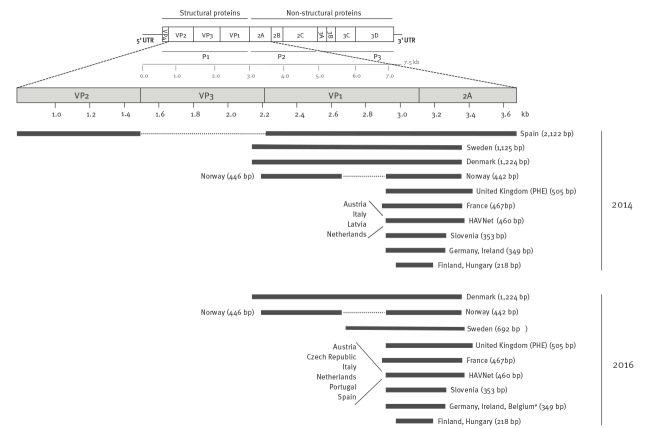
Hepatitis A virus genome and targets used for sequence-based typing, European Union/European Economic Area countries, 2014 (n = 15 countries providing information) and 2016 (n = 17 countries providing information)

The sequenced genome regions and their length are shown in Figure 3. All of the responding EU/EEA countries (n = 15/15 in 2014 and n = 17/17 in 2016) sequence the VP1/2A junction but the sequence length varies, with a minimum overlap of 218 nt for both years. Three countries (Denmark, Norway; Sweden before 2016) sequence the 5’ end of VP1 in addition to the VP1/2A junction. One country (Spain) used the VP1 and VP2 region in addition to VP1/2A for amplification before 2016. Four countries used the HAVNET protocol in 2014 and the numbers increased to six in 2016.

bp: base pair; kb: kilo bases; PHE: Public Health England; ssRNA: single stranded RNA; UTR: untranslated region; VP: viral protein.

^a^ Belgium changed the region of the genome targeted for amplification/sequencing after 2016 and now uses the HAVNET protocol.

The ( + ) ssRNA (7.5 kb) consists of three open reading frames (P1–P3) encoding structural (P1) and non-structural proteins (P2, P3). P1 encodes the capsid polypeptides (VP1–4), and the P2 and P3 regions encode the non-structural polypeptides required for virus replication. The horizontal bars represent the position in the genome of the region amplified and sequenced by the respective countries in 2014 and 2016. The sizes of the respective regions after removing primer binding sites are indicated in parentheses next to the horizontal bars. The GenBank sequence NC_001489 (HAV, complete genome) was used as reference.

### Collaborations with the food sector and HAVNET

**Figure 4:**
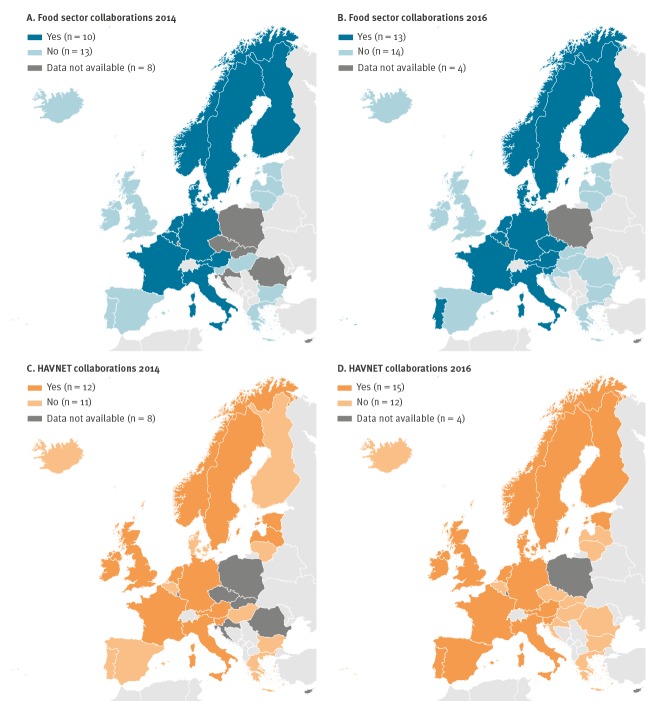
Collaborations of the European Union/European Economic Area countries with the (A, B) food sector and (C, D) HAVNET in 2014 (n = 23 countries providing information) and 2016 (n = 27 countries providing information)

Ten of 23 countries reported collaborations with the food sector for comparing sequences in 2014 and 13 of 27 in 2016. Portugal and Slovenia started to collaborate with the food sector after the 2014 survey. Of the countries joining the survey in 2016, the Czech Republic reported comparing sequences with the food sector (Figure 4A and B). Belgium stated that this is not done on a routine basis but only for outbreak investigations.

Countries collaborating with the food sector (food safety authorities and reference laboratories for food) on comparing sequences obtained from humans and/or food samples (A) in 2014 and (B) 2016 and countries collaborating with HAVNET (global network of hepatitis A laboratories for sharing molecular and epidemiological data on hepatitis A - www.havnet.nl) in depositing and/or comparing data in the HAVNET database (C) in 2014 and (D) 2016.

The number of countries reporting collaborations with HAVNET increased between the two surveys in 2014 (n = 12/23) and 2016 (n = 15/27). Latvia reported collaboration with HAVNET in 2014, but no longer in 2016. Denmark, Finland, Portugal and Spain started collaborating with HAVNET after 2014 (Figure 4C and D).

The majority of the countries reporting collaboration with HAVNET in 2016 submitted sequences to the database or used it for comparison of sequences. Of the countries submitting sequences, only three indicated a regular sequence submission (the Netherlands, Portugal and Sweden). The remaining countries only submitted in case of outbreak suspicion or on a non-regular basis (Austria, Finland, Germany, Ireland, Norway, and the UK). Five countries provided an answer about the time delay between sequencing and submission of results to HAVNET, Sweden stated that submission of sequences occurred immediately after results were obtained. For the other four replying countries (Germany, Norway, Portugal, and the UK), the time delays differed significantly, ranging from 30 days to several years, depending on the country capacities and prioritisations.

## Discussion

The two surveys performed in 2014 and 2016 provide insights into the molecular surveillance and sequencing practices within the EU/EEA and assess the changes introduced following a large food-borne outbreak in 2013–14. Joint practices such as a common target region for sequencing suggested in the ECDC multidisciplinary HAV expert meeting in 2014, allow the comparison of results and therefore provide information for action. Our analysis identified some progress in this respect between 2014 and 2016, with most countries reporting positive developments in different aspects of sequencing practices. In total, 13 procedural changes were observed, indicating an improvement in preparedness and outbreak response capacity in Europe (Figure 1): the number of countries with a central collection and storage of human HAV samples increased, as did the countries with a mandatory referral system, those performing sequencing, and those collaborating with the food sector and HAVNET. According to the replies in 2014 and 2016, sequence-based typing of HAV samples has been implemented in the majority of EU/EAA countries, except for most eastern EU countries. It is important to note that six EU/EEA countries (Croatia, Estonia, Iceland, Latvia, Lithuania, Malta) report a mean of < 20 confirmed hepatitis A cases per year in the period 2014–16, which makes the implementation of sequencing not a priority for these countries [6]. Only four countries did not provide information in both surveys.

One of the most important findings is that there is capacity for sequencing in the majority of the EU/EEA countries allowing epidemiologically meaningful comparison of results with all countries performing sequencing of an overlapping region of the HAV genome (Figure 3).

Although reporting of hepatitis A was compulsory in all the 23 countries responding to the survey in 2014, systematic surveillance of HAV infections could be further improved in the majority of EU/EEA countries. The absence of a centralised sample collection or a referral system (in 12 and 13 countries in 2016, respectively) makes it challenging to compare HAV sequences, even on the national level.

The crucial role of sequence data analysis to investigate outbreaks and define transmission pathways has been demonstrated several times [10,19,30]. With a hepatitis A incubation period of about 1 month, it is challenging to link apparently sporadic cases and identify transmission mechanisms and/or vehicles of infection based only on patients’ interviews. Sequencing analysis of HAV isolated from patients supports linking cases, revealing infection routes and identifying the vehicle of infection in food-borne outbreaks. In 2013, Italy was the most affected EU/EEA country in a large food-borne hepatitis A outbreak with > 1,200 reported cases. Sequencing was described as having a key role in delineating this outbreak, linking cases lacking any epidemiological link and identifying the outbreak strain in the suspected food vehicle. Sequencing also revealed the circulation of other unrelated strains [20]. Greece performed sequencing during a hepatitis A outbreak among refugees, asylum seekers and migrants in 2016, demonstrating that cases were imported and not locally acquired [31]. In 2018, sequencing revealed a link between cases in Sweden and Austria during an HAV outbreak and confirmed imported frozen strawberries as the vehicle of infection in Sweden. Consequently, the strawberries were withdrawn from the market, which led to the end of the outbreak in Sweden [32]. The importance of a routine surveillance system, including prompt referral and typing of HAV samples, was also highlighted in a Dutch study published in 2014 [33]. The authors pointed out that large outbreaks are rapidly detected because of the large number of patients, but for the detection of slowly spreading and dispersed clusters molecular typing is essential. In addition to providing links between cases otherwise not recognised, sequencing helps to distinguish sporadic cases from outbreak cases, which improves the efficiency of epidemiological studies, and is essential to verify the end of an outbreak. Nevertheless, HAV sequence analysis is most useful to support outbreak investigations when applied together with relevant associated epidemiological information.

Regarding the genomic region to be sequenced, protocols for HAV sequence-based typing differ within the EU/EEA region, with all responding countries sequencing at least a part of the VP1/2A junction (minimum fragment of 218 nt). The overlapping fragment in the same selected genome region allows a minimal comparison of different strains between countries. The VP1/2A junction is known to be one of the most variable regions of the HAV genome and therefore allows good resolution when comparing sequences. In the early 1990s, the different HAV genotypes were initially defined based on a 168-nt fragment of the VP1/2A junction [23] but were re-classified in the beginning of 2000 based on the longer VP1 sequence (900 nt) [34]. Some HAV strains differ from each other in > 20% of the nt in the VP1/2A region and studies demonstrated a correlation of genotype and subgenotype with geographical clustering [23,35,36]. Due to the high variability of this region and despite the short size (ca 500 nt), genotyping results can be obtained and genetic relatedness among HAV strains determined [36]. For outbreak investigations, analysis of the variable VP1/2A sequence together with the epidemiological association is sufficient to follow transmission events [37].

In order to achieve more meaningful molecular typing support for surveillance of hepatitis A in Europe and to be able to compare HAV strains circulating in different countries, sequencing protocols targeting the same region are required. The short target of the VP1/2A junction allows easy and fast sequencing at lower cost compared with sequencing of larger regions. Furthermore, it is the most commonly investigated region in outbreaks, allowing the comparison of large numbers of previously sequenced strains in public databases [20].

Analysis of sequencing data also offers substantial benefits for identification of the outbreak vehicle or source of infection. Since HAV infections are often food-borne, sequencing of food items and collaborations with the food sector are of paramount importance. Most (13/17) countries performing sequencing in 2016 indicated collaboration with the food sector and comparison of sequences detected in human and food samples. Although sequencing from food samples is challenging due to the low viral load, inefficient extraction from different food matrices, the long incubation period of hepatitis A (up to 6 weeks) and the resulting recall bias of food consumption, it is invaluable in tracing the source of infections and providing evidence for public health actions [33,38]. A large part of the eastern European countries, which report the majority of the hepatitis A cases in the EU, neither perform sequencing yet, nor have collaborations established with the food sector, with consequent limited knowledge of the strains circulating in those countries.

HAVNET provides a global database that offers good support for outbreak investigations, e.g. comparison of strains at national and EU/EEA level. The network consists of virologists from universities and public health institutes using an online password-protected database platform. The use of the database is on a ‘give-and-take-basis’ and involves the signing of a confidentiality agreement. The platform provides online analysis and visualisation tools such as basic local alignment search tool (BLAST), phylogeny and a geographical analysis tool [25]. So far, HAVNET offers the only centralised collection of sequencing data and a standard protocol for HAV sequencing from human samples which additionally enables the comparison with HAV strains from food samples. Molecular data can be exchanged, strains circulating in different countries rapidly identified and therefore outbreak responses to multi-country threats improved. Although HAVNET provides a useful tool for outbreak investigations, only 15 countries reported collaborations with HAVNET in 2016.

To our knowledge, this is the first time information has been systematically gathered on the variety of existing laboratory practices supporting hepatitis A surveillance within the EU/EEA. Study limitations include the survey participation: despite a good participation, four countries did not reply to both surveys. Although some countries did not have sequencing capacity, they reported having access to it or collaborating with other countries during hepatitis A outbreaks. Greece, Latvia, Luxembourg and Malta performed sequencing in 2017 in the context of the hepatitis A outbreak mostly affecting MSM [15]. In Estonia, samples were referred to another laboratory outside the country (National Institute for Public Health and the Environment in the Netherlands) and Bulgaria sent its samples for sequencing to the Istituto Superiore di Sanità in Italy for ad hoc scientific collaborations [39]. A further limitation is that the question about the sequencing protocol was asked in 2014 only and the typing protocol may be different nowadays. However, countries had the opportunity to indicate changes in their sequencing practices and for the countries indicating to have started sequencing after 2014, additional information was collected separately. Spain decided to sequence 460 nt of the VP1/2A junction (corresponding to the HAVNET protocol) instead of complete VP1, VP2 and 2A after 2016 due to the high number of cases related to the European HAV outbreak disproportionally affecting MSM [15]. Sweden changed its protocol after the expert meeting in 2014 to cover the 460 nt fragment suggested in the HAVNET protocol plus 232 nt of the 3’ VP1 region. Belgium replaced its sequencing protocol with the HAVNET protocol after 2016. Therefore, the results might slightly underestimate the true sequencing capacity to support hepatitis A surveillance in the EU/EEA.

In conclusion, the surveys highlighted that many of the EU/EEA countries perform sequencing with rather variable procedures, but their sequenced regions are overlapping and thus comparable for surveillance purposes. Nonetheless, European collaboration in sharing and comparing HAV sequences on circulating strains in a timely manner can be further strengthened. Resulting collaborations could contribute significantly to accelerate public health responses and thereby stop transmission chains across borders and reduce the hepatitis A incidence. The population susceptibility to HAV infection in the EU/EEA has increased due to improved hygiene and sanitation that, together with the availability of a safe and effective vaccine, result in decreased virus circulation. However, hepatitis A surveillance in the EU/EEA is essential since the high population susceptibility in most of the countries, coupled with the high population mobility and single food market, increase the potential for cross-borders outbreaks. HAV sequencing is necessary to ensure the most efficient and timely response to such threats across the EU.

The application of whole genome sequencing (WGS) has increased within recent years and has become the reference molecular typing method in outbreak investigations [40]. Applying WGS to the detection of pathogens provides several advantages such as fast turn-around time and reduction of costs and workload. A high discrimination between HAV strains can be achieved since WGS allows the detection of minor variants. In the coming years, WGS might replace the sequencing of the VP1/2A region. In the meantime, a higher degree of harmonisation in amplifying the VP1/2A region will help to improve preparedness and accelerate public health response in Europe.

## Supplementary Data

449000 Supplement 1

## Supplementary Data

461000 Supplement 2
