# Supplementary material for: Improving preparedness to respond to cross-border hepatitis A outbreaks in the European Union/European Economic Area: towards comparable sequencing of hepatitis A virus
Source: Euro Surveill. 2019 Jul 11;24(28):1800397. doi: 10.2807/1560-7917.ES.2019.24.28.1800397 (PMC6636214; doi:10.2807/1560-7917.ES.2019.24.28.1800397)
Supplement: Supplement 1 [file 1800397_Enkirch_Supplement1.pdf]

Supplement 1

This supplementary material is hosted by *Eurosurveillance* as supporting information alongside the article '**Improving preparedness to respond to cross-border hepatitis A outbreaks in the European Union/European Economic Area: towards comparable sequencing of hepatitis A virus**' on behalf of the authors who remain responsible for the accuracy and appropriateness of the content. The same standards for ethics, copyright, attributions and permissions as for the article apply. *Eurosurveillance* is not responsible for the maintenance of any links or email addresses provided therein.

Rapid consultation on hepatitis A virus characterization - 2014

Fields marked with * are mandatory.

Purpose of the rapid consultation

Recent foodborne outbreaks of Hepatitis A virus (HAV) affecting multiple European Member States highlight the importance of comparability of molecular characterization of HAV across public health sectors. However, protocols for HAV molecular typing from human samples, when existing, proved to be heterogeneous amongst European MS.

The aim of this rapid consultation in EU/EEA Member States is to get a snapshot of present HAV genotyping practices and protocols, and to provide a summary of the situation for the discussion in the HAV expert consultation meeting at ECDC on 23-24 October 2014. The summary of this consultation and the outcome of the expert meeting will be sent to the European FWD network for information.

We kindly ask you to reply to this consultation or forward the link to the relevant expert at the national level. We expect to have only one reply per country.

The estimated time to reply to the questions is max 15 minutes. If you have any questions please contact us at fwd@ecdc.europa.eu.

We thank you very much in advance for your contribution!

Ettore Severi, Katrin Leitmeyer and Johanna Takkinen
On behalf of ECDC FWD team

Respondents background

* Country

* Name and email of the respondent

Hepatitis A - virus characterization practices and protocols

* 1. Does your country (public health institute or collaborating centre) collect and store diagnostic samples of hepatitis A cases centrally?

- Yes
- No

1a. If yes, please specify (e.g. which samples, where etc.)

*2. Does your country have a referral system for diagnostic confirmation of hepatitis A samples?

- Yes
- No

2a. If yes, please describe shortly the system

*3. Does your country (public health reference laboratory or collaborating centre) perform sequence-based typing of hepatitis A virus?

- Yes
- No

3a. If yes, please specify for which purposes:

- [ ] For surveillance (routinely)
- [ ] For outbreak detection and investigation
- [ ] For research

3a. If yes, please specify for which purposes

*3b. If no, do you refer samples for sequencing to any other laboratory or company?

- Yes
- No

3b1. If yes, please specify

*4. Do you collaborate with food sector on comparing sequences obtained from humans and/or food samples?

- Yes
- No

*5. Does your country (public health institute or collaborating centre) collaborate with/deposit/compare data in the HAVNet database? (http://www.rivm.nl/en/Topics/H/HAVNET)

- Yes
- No

Free comments and feedback to ECDC
