# Supplementary material for: Improving preparedness to respond to cross-border hepatitis A outbreaks in the European Union/European Economic Area: towards comparable sequencing of hepatitis A virus
Source: Euro Surveill. 2019 Jul 11;24(28):1800397. doi: 10.2807/1560-7917.ES.2019.24.28.1800397 (PMC6636214; doi:10.2807/1560-7917.ES.2019.24.28.1800397)
Supplement: Supplement 2 [file 1800397_Enkirch_Supplement2.pdf]

Supplement 2

This supplementary material is hosted by *Eurosurveillance* as supporting information alongside the article '**Improving preparedness to respond to cross-border hepatitis A outbreaks in the European Union/European Economic Area: towards comparable sequencing of hepatitis A virus**' on behalf of the authors who remain responsible for the accuracy and appropriateness of the content. The same standards for ethics, copyright, attributions and permissions as for the article apply. *Eurosurveillance* is not responsible for the maintenance of any links or email addresses provided therein.

Rapid consultation on hepatitis A virus characterization - 2016

Fields marked with * are mandatory.

Purpose of the rapid consultation

In 2013 and 2014, large foodborne outbreaks of Hepatitis A virus (HAV) affecting multiple European Member States (MS) highlighted the importance of comparability of molecular characterization of HAV across public health sectors. However, protocols for HAV molecular typing from human samples, when existing, proved to be heterogeneous amongst European MS.

In summer 2014, ECDC performed a rapid consultation among the EU/EEA MSs collecting information on HAV genotyping practices and protocols. The results of the consultation provided a snapshot of the situation in 2014 and built the case for the discussion in the HAV expert consultation meeting at ECDC on 23-24 October 2014. The meeting participants agreed to recommend a standard protocol for HAV sequencing as proposed by HAV-NET.

ECDC is now proposing a second consultation to monitor progress and make information on the EU level preparedness for HAV outbreak investigations available to the scientific community. The summary of this consultation and the comparison with the previous one will be shared with the European FWD network (FWD-Net) and submitted to a peer-reviewed journal. We invite each responding country to identify up to two co-authors for the publication.

We kindly ask you to reply to this short consultation or forward the link to the relevant expert at the national level. We expect to have only one reply per country. Thank you for replying **by 30 September 2016**.

The estimated time to reply to the questions is max 20 minutes. If you have any questions please contact us at fwd@ecdc.europa.eu.

We thank you very much in advance for your contribution!

Ettore Severi, Katrin Leitmeyer and Johanna Takkinen

Respondents background

* Country

* Name and email of the respondent

Hepatitis A - virus characterization practices and protocols

* 1. Does your country (public health institute or collaborating centre) collect and store diagnostic samples of hepatitis A cases centrally?

- ○ Yes
- ○ No

1a. If yes, please specify (e.g. which samples, where etc.)

* 2. Does your country have a referral system for diagnostic confirmation of hepatitis A samples?

- ○ Yes
- ○ No

2a. If yes, please describe shortly the system

* 3. Does your country (public health reference laboratory or collaborating centre) perform sequence-based typing of hepatitis A virus?

- ○ Yes
- ○ No

3a. If yes, please specify for which purposes:

- ☐ Routine surveillance
- ☐ Outbreak detection and investigation
- ☐ Research
- ☐ Other purposes

3a1. If for other purposes, please specify which:

* 3b. If no, do you refer samples for sequencing to any other laboratory or company?

- ○ Yes
- ○ No

3b1. If yes, please specify which laboratory

* 4. Do you collaborate with the food sector on comparing sequences obtained from humans and/or food samples?

- ○ Yes

- No

*5. Does your country (public health institute or collaborating centre) collaborate with/submit/compare data in the HAVNet database? (http://www.rivm.nl/en/Topics/H/HAVNET)

- Yes
- No

5a. If yes, how do you collaborate?

5b. If yes, do you submit sequences to the HAVNET database?

- Yes
- No

5c. If yes,do you submit sequences on regular basis or on *ad hoc* basis?

- [ ] Regular basis
- [ ] When an outbreak is suspected
- [ ] On request
- [ ] On other *ad hoc* basis

5d. Please specify:

5e. If yes, which is the median delay (in calendar days) and the minimum-maximum range between sampling date and date of submission to HAVNET for the sequences you have submitted in 2015?

Free comments and feedback to ECDC
